# Efficacy and Safety of Moderate-Dose Stereotactic Body Radiation Therapy (SBRT) for Patients with Oligometastatic Lung Cancer Unfit for Standard SBRT: A Preliminary Single-Center Study

**DOI:** 10.3390/jcm15166337

**Published:** 2026-08-16

**Authors:** Jaehyeon Park, Bong Kyung Bae, Min Kyu Kang, Jongmoo Park

**Affiliations:** 1 Department of Radiation Oncology, Yeungnam University College of Medicine, Daegu 42415, Republic of Korea; drjhyeon@ynu.ac.kr; 2 Department of Radiation Oncology, School of Medicine, Kyungpook National University, Daegu 41944, Republic of Korea; bae8808@knu.ac.kr (B.K.B.); mkkang@knu.ac.kr (M.K.K.)

**Keywords:** stereotactic body radiation therapy, oligometastatic disease, lung cancer, moderate-dose radiotherapy, local control, lymph node metastasis

## Abstract

**Background/Objectives**: Stereotactic body radiation therapy (SBRT) is increasingly used for oligometastatic lung cancer. However, standard ablative SBRT (BED_10_ of 100 Gy or higher) is not feasible when lesions abut critical organs or patients have poor performance status. This study aimed to evaluate the efficacy and safety of moderate-dose SBRT (BED_10_ below 100 Gy but exceeding conventional palliative doses) in patients with oligometastatic lung cancer unfit for standard ablative SBRT. **Methods**: We retrospectively analyzed patients treated at a single institution between October 2019 and March 2025. Eligible patients had pathologically confirmed lung cancer with extracranial oligometastasis and received 25–40 Gy in 4–7 fractions. Patients with brain or bone metastases were excluded. The primary endpoint was local control. Secondary endpoints were event-free survival, disseminated disease-free survival, overall survival, and treatment-related toxicity. **Results**: Forty-three patients with 55 lesions were analyzed. The mean age was 66 years, and lymph node metastases were the most frequent site (69.1%). The most common regimen was 35 Gy in 5 fractions (63.6%). At a median follow-up of 14.7 months, objective response (complete or partial) was achieved in 54 of 55 lesions (98.2%). The 1-year and 2-year local recurrence-free survival rates were 89.7% and 86.9%, respectively, and the 1-year and 2-year overall survival rates were 83.2% and 56.1%, respectively. Local control differed by metastatic site, with 2-year rates of 95.5% for lymph node lesions and 56.2% for lesions of the lung and chest wall (*p* = 0.002), whereas smaller differences were observed by BED_10_ and oligometastatic subtype. Treatment-related toxicities occurred in 8 of 43 patients (18.6%), all of grade 1–2, and radiation pneumonitis was the most common (9.3%). No grade 3 or higher events occurred. **Conclusions**: In this preliminary single-center experience, moderate-dose SBRT achieved favorable local control with acceptable toxicity in oligometastatic lung cancer patients unfit for standard ablative SBRT, supporting a risk-adapted approach.

## 1. Introduction

Oligometastatic disease (OMD) is considered an intermediate state between localized cancer and widespread metastatic disease, in which a limited metastatic burden may be amenable to curative-intent or durable disease-controlling local therapy in selected patients [1,2]. Although definitions vary, contemporary consensus documents generally describe OMD as ≤3–5 lesions or ≤5 metastatic sites for which metastasis-directed local therapy may contribute to improved disease control in selected patients. In lung cancer, particularly non-small cell lung cancer (NSCLC), the reported incidence of OMD varies substantially according to definition, staging intensity, and timing of assessment, ranging from approximately 16–33% at diagnosis to approximately 7–50% over the disease course, including oligorecurrent and oligoprogressive states [3,4,5]. Recent improvements in systemic therapy have been accompanied by a redefinition of the therapeutic role of radiotherapy in metastatic NSCLC. In selected patients with limited metastatic burden, radiotherapy is increasingly employed as a metastasis-directed modality aimed at durable local control and prolongation of progression-free intervals rather than solely symptom palliation. Stereotactic body radiotherapy (SBRT) is widely used as a metastasis-directed modality for oligometastatic lung cancer because it delivers highly conformal high-dose radiation over a short treatment course. Prospective and randomized studies support local consolidative strategies incorporating SBRT in appropriately selected patients. In oligometastatic NSCLC, randomized trials have demonstrated improved progression-related outcomes with local consolidative therapy compared with systemic therapy alone, supporting the rationale for metastasis-directed approaches [6,7].

Despite its increasing use, the optimal dose fractionation for SBRT in metastatic disease remains uncertain. Many reports and practice patterns favor “ablative” regimens, commonly defined as a biologically effective dose (BED_10_) ≥ 100 Gy, because of their association with high local control (LC) [7]. However, delivery of ablative SBRT is often challenging in routine practice due to organ-at-risk constraints [8,9,10], competing systemic treatment requirements, or patient-related factors such as poor performance status. These considerations support a tailored approach to SBRT dose selection. Moderate-dose regimens could be considered a potential alternative that balances efficacy and safety in patients for whom fully ablative treatment is not feasible. In particular, lymph node metastases, which constitute a substantial proportion of oligometastatic NSCLC, are often located adjacent to critical thoracic structures, where strict adherence to ablative dose thresholds may compromise organ-at-risk constraints and treatment feasibility. However, evidence supporting the efficacy and safety of moderate-dose SBRT remains limited, particularly in lung cancer-specific cohorts [11,12]. Patients in whom ablative doses cannot be safely delivered are unlikely to be represented in prospective trials that require ablative dose prescription, and outcomes in this population have been less well characterized. In addition, published series have largely addressed either ablative SBRT or conventional palliative radiotherapy, leaving the intermediate dose range relatively under-reported.

Accordingly, this preliminary single-center study aimed to evaluate the efficacy and safety of moderate-dose SBRT in patients with oligometastatic lung cancer who are unfit for standard ablative SBRT because of poor performance status or proximity of metastatic lesions to critical structures.

## 2. Materials and Methods

### 2.1. Patients

This retrospective study analyzed patients treated with moderate-dose SBRT with a BED_10_ < 100 Gy between October 2019 and March 2025 at a single institution. The study protocol was approved by the Institutional Review Board.

Eligible patients were aged ≥19 years and had pathologically confirmed lung cancer, including NSCLC and small cell lung cancer (SCLC), with extracranial OMD identified on CT and/or PET/CT. OMD was defined as ≤5 metastatic lesions involving <3 organs. Oligometastatic states were classified according to the ESTRO/EORTC consensus [1]. De novo OMD was defined as the first diagnosis of oligometastatic disease without prior history of metastasis. Repeat OMD was defined as prior oligometastatic disease with subsequent re-presentation as oligometastatic. Induced OMD was defined as prior polymetastatic disease that converted to an oligometastatic state, typically following systemic therapy. All three categories were eligible for inclusion in the present study. Confirmation of active oligometastatic disease prior to SBRT was carried out according to the patient’s OMD state. In patients with de novo OMD, oligometastatic lesions were identified during the initial staging workup, which routinely included whole-body 18F-FDG PET-CT; no additional imaging for active disease confirmation was therefore required. In patients with clinical or radiographic findings suggestive of a synchronous double primary tumor rather than metastatic disease, tissue biopsy was performed when the lesion was anatomically accessible. In patients with repeat or induced OMD, lesions were considered active if they showed an increase in size compared with prior imaging according to Response Evaluation Criteria in Solid Tumors (RECIST) version 1.1 [13], or if they represented newly developed lesions. In such cases, 18F-FDG PET-CT was performed to confirm metabolic activity when clinically feasible; in patients for whom PET-CT could not be obtained, assessment was based on CT imaging alone. In accordance with the 2023 ASTRO/ESTRO clinical practice guideline, oligometastatic status was confirmed by imaging rather than by per-lesion histological confirmation.

Patients with brain or bone metastases were excluded. There were no restrictions based on prior systemic therapy, and all patients had an Eastern Cooperative Oncology Group (ECOG) performance status of 0–1. Only patients treated with alternative moderate-dose SBRT were included, defined as hypofractionated regimens with a BED_10_ exceeding conventional palliative doses but <100 Gy. Standard palliative regimens (e.g., 20 Gy in 5 fractions or 30 Gy in 10 fractions) were excluded.

### 2.2. Radiotherapy

All patients underwent CT simulation. Four-dimensional computed tomography (4D-CT) was performed for lesions affected by respiratory motion, including pulmonary lesions and abdominal lymph nodes (LNs). CT images were acquired with a slice thickness ≤ 5 mm. The gross tumor volume (GTV) was defined as the visible metastatic lesion. For 4D-CT, the GTV was contoured on each respiratory phase and combined to generate the internal target volume (ITV). The planning target volume (PTV) was created by adding a uniform 5-mm margin to the GTV or ITV.

Moderate-dose SBRT regimens were selected at the discretion of the treating physician based on tumor location and patient condition, with doses intentionally exceeding standard palliative levels. Respiratory-gated radiotherapy (RT) was used when appropriate. RT was delivered once daily using volumetric-modulated arc therapy or intensity-modulated RT with photon energies of 6–10 MV. Image guidance with cone-beam CT was performed before each fraction.

### 2.3. Follow-Up and Outcome Evaluation

Initial tumor response was assessed using CT at 1 month after completion of SBRT according to RECIST version 1.1. Patients were subsequently followed every 3 to 6 months, with imaging studies—primarily contrast-enhanced CT—selected according to clinical context and treated site.

LC was defined as the absence of radiographic progression within the PTV. Secondary endpoints included event-free survival (EFS), disseminated disease-free survival (DDFS), overall survival (OS), and treatment-related toxicity. Disseminated disease was defined as the development of >5 new or progressive metastatic lesions at progression. Survival intervals were calculated from the completion of SBRT to the relevant event or last follow-up. Treatment-related toxicities were graded using the Common Terminology Criteria for Adverse Events, version 5.0.

### 2.4. Statistical Analysis

Patient characteristics were summarized using descriptive statistics. LC, PFS, DDFS, and OS were estimated using the Kaplan–Meier method, and 95% confidence intervals (CIs) were calculated for survival estimates. Local control was additionally compared across prespecified strata, namely metastatic site (lymph node, lung and chest wall, and adrenal gland), BED_10_ (≥59.5 Gy, corresponding to 35 Gy in 5 fractions, versus <59.5 Gy), and oligometastatic subtype (de novo, repeat, and induced OMD), using the log-rank test. Analyses of local control were performed at the lesion level, whereas analyses of overall survival were performed at the patient level. Univariable Cox proportional hazards regression was used to examine factors associated with local recurrence, and variables with *p* < 0.2 on univariable analysis were entered into a multivariable model; hazard ratios (HRs) with 95% CIs were reported. All variables had complete data, and no imputation was performed. A *p*-value < 0.05 was considered statistically significant. Analyses were conducted using R software (version 4.4.2; R Foundation for Statistical Computing, Vienna, Austria).

## 3. Results

A total of 43 patients with 55 oligometastatic lesions were included in the analysis. The baseline patient and disease characteristics are summarized in Table 1. Most patients (*n* = 34, 79.1%) had NSCLC, while nine (20.9%) had SCLC. Among patients with NSCLC, adenocarcinoma was the most common histologic subtype (*n* = 19, 44.2%), followed by squamous cell carcinoma (*n* = 9, 20.9%). Most patients (79.1%) had been diagnosed with primary lung cancer > 6 months before SBRT. At the time of SBRT, 86.0% of lesions showed radiographic progression, and 69.8% of patients were receiving systemic therapy.

At that time, 14.0% of patients were treated for oligometastatic lesions representing the first metastatic occurrence (de novo oligometastasis). The remaining 86.0% had prior metastasis: 27.9% had previously presented with OMD (repeat oligometastasis), and 58.1% had a history of widespread metastasis (induced oligometastasis). Most patients (79.1%) had a single treated lesion, while six (14.0%) had two lesions and three (7.0%) had three lesions. Of 55 lesions treated in 43 patients, 38 (69.1%) were lymph-node metastases and 17 (30.9%) were non-lymph-node lesions in the lung, adrenal gland, or chest wall (Table 2).

Radiation doses ranged from 25 to 40 Gy (median, 35 Gy) in 4–7 fractions (median, 5 fractions). The most common regimen was 35 Gy in 5 fractions, administered to 35 lesions (63.6%). All treatments were delivered once daily using VMAT or IMRT with image guidance. Respiratory-gated RT was employed for selected lesions subject to respiratory motion, particularly in the lower lung lobes or abdominal LNs.

Tumor response was evaluated 1 month after SBRT according to RECIST version 1.1. Partial response (PR) was observed in 51 lesions (92.7%), complete response in three (5.5%), and stable disease in one (1.8%). No cases of progressive disease were observed.

**Figure 1:**
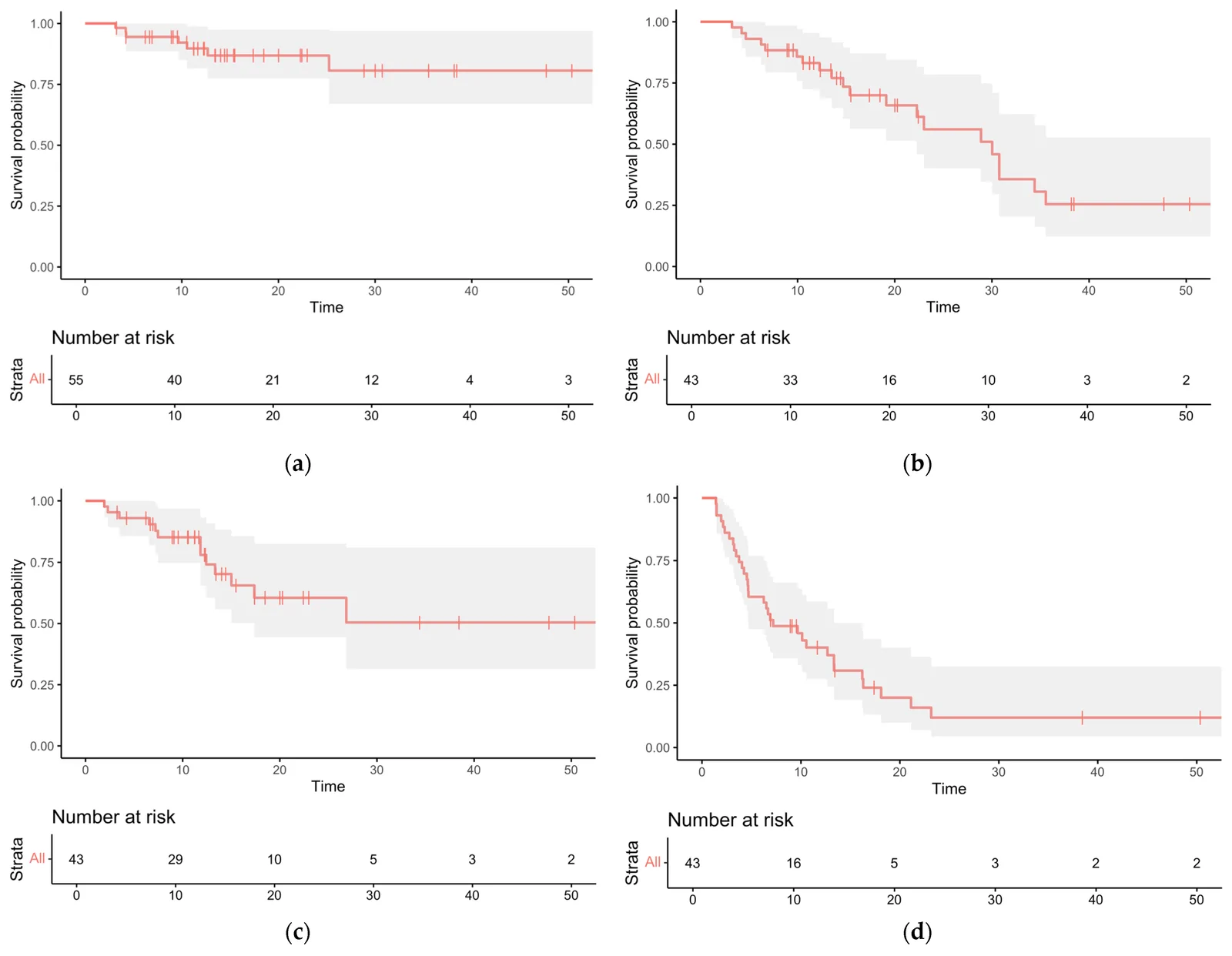
Kaplan–Meier curves for (**a**) local recurrence-free survival, (**b**) overall survival, (**c**) freedom from widespread dissemination, and (**d**) event-free survival in patients with oligometastatic lung cancer treated with moderate-dose SBRT.

Median follow-up was 14.7 months (range, 3.3–54.0 months). A total of 7 local recurrence events were observed during follow-up, of which 2 occurred in lymph node lesions and 5 in non-lymph node lesions. Local recurrence-free survival (LRFS) at 1 and 2 years was 89.7% (95% CI, 81.5–98.8) and 86.9% (95% CI, 77.4–97.5), respectively (Figure 1a). At the time of analysis, 20 of 43 patients (46.5%) had died. Median overall survival was 30.0 months, and OS at 1 and 2 years was 83.2% (95% CI, 64.1–89.6) and 56.1% (95% CI, 30.4–68.3), respectively (Figure 1b). Widespread dissemination occurred in 13 patients, and freedom from widespread dissemination was 78.0% (95% CI, 60.3–88.6) at 1 year and 60.5% (95% CI, 25.5–70.9) at 2 years (Figure 1c). A total of 33 events, comprising disease progression or death, were observed. Median event-free survival (EFS) was 7.2 months, and EFS at 1 and 2 years was 40.1% (95% CI, 22.3–51.8) and 12.0% (95% CI, 3.4–26.6), respectively (Figure 1d). The patterns of failure were predominantly extracranial and systemic: in-field recurrence occurred in 7 patients, whereas out-of-field progression and distant dissemination were observed in 26 and 13 patients, respectively.

**Figure 2:**
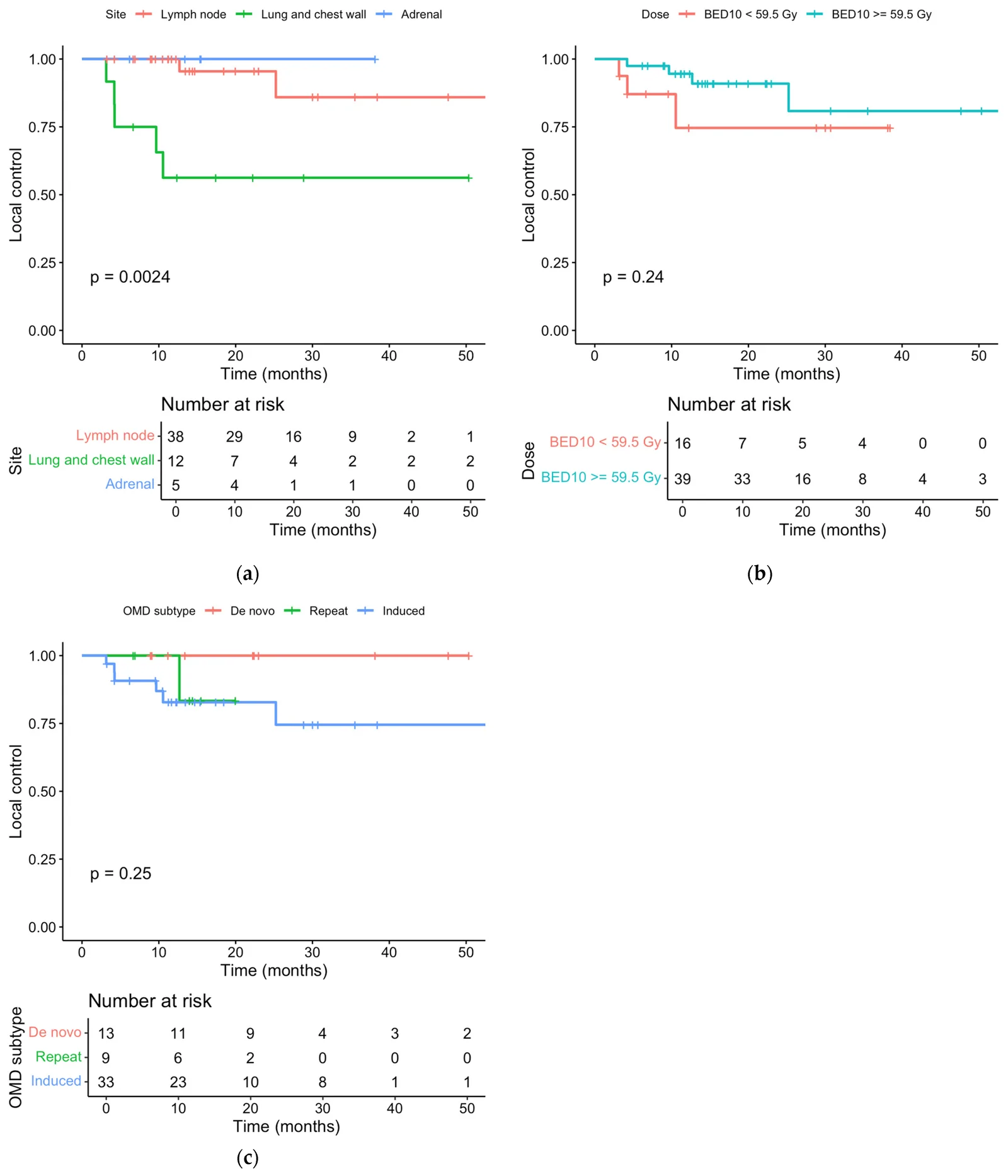
Kaplan–Meier curves for local control stratified by (**a**) metastatic site (lymph node vs. non-lymph node), (**b**) BED_10_ (≥59.5 Gy vs. <59.5 Gy), and (**c**) oligometastatic disease subtype in patients with oligometastatic lung cancer treated with moderate-dose SBRT.

Local control differed significantly by metastatic site (Table 3, Figure 2a). For this analysis, the two chest-wall lesions were grouped with pulmonary lesions. The 2-year local control rate was 95.5% (95% CI, 87.1–100) for lymph node lesions, 56.2% (95% CI, 33.6–94.3) for lesions of the lung and chest wall, and 100% for adrenal lesions (log-rank *p* = 0.002). Local control was numerically higher for lesions treated with a BED10 of 59.5 Gy or more than for those treated with a lower dose, with 2-year rates of 90.9% (95% CI, 81.5–100) and 74.6% (95% CI, 52.1–100), respectively, although this difference was not statistically significant (log-rank *p* = 0.245, Figure 2b). By oligometastatic subtype, no in-field recurrence occurred among the 13 lesions treated in patients with de novo OMD, whereas the 2-year local control rate was 82.8% (95% CI, 70.0–98.0) for induced OMD (log-rank *p* = 0.247, Figure 2c). Overall survival also differed by oligometastatic subtype. Death occurred in 2 of 10 patients with de novo, 1 of 8 with repeat, and 17 of 25 with induced OMD. The 2-year overall survival rate was 62.5% (95% CI, 32.0–100) for de novo and 48.2% (95% CI, 30.5–76.4) for induced OMD, whereas the estimate for repeat OMD was not evaluable at 2 years because of limited follow-up (log-rank *p* = 0.050).

On univariable Cox regression, non-lymph node location was associated with local recurrence (HR 6.008; 95% CI, 1.165–30.976; *p* = 0.032), and age of 65 years or older (HR 5.343; 95% CI, 0.992–28.772; *p* = 0.051) and a history of polymetastatic disease (HR 4.327; 95% CI, 0.517–36.242; *p* = 0.177) showed borderline associations (Table 4). In the multivariable model including these three variables, none retained statistical significance, and all confidence intervals were wide (non-lymph node location: HR 4.257; 95% CI, 0.645–28.100; *p* = 0.133; age of 65 years or older: HR 4.151; 95% CI, 0.516–33.380; *p* = 0.181; history of polymetastatic disease: HR 8.219; 95% CI, 0.890–75.880; *p* = 0.063).

Treatment-related toxicities occurred in 8 of 43 patients (18.6%), all grade 1–2, with no grade ≥ 3 events (Table 5). Radiation pneumonitis was the most common, observed in 4 patients (9.3%).

## 4. Discussion

In this retrospective study of 43 patients with 55 oligometastatic lung cancer lesions treated with moderate-dose SBRT (BED_10_ below 100 Gy but exceeding conventional palliative doses; median, 59.5 Gy), we observed favorable LC with acceptable safety. At a median follow-up of 14.7 months, local control remained high through 2 years, whereas overall and disseminated disease-free survival declined progressively. The steep decline in event-free survival indicates that failure was driven by systemic rather than local progression. Data remain limited for patients in whom moderate doses were selected because ablative dosing was not feasible, and our cohort may help to characterize outcomes in this setting. Reporting results across this intermediate dose range may also complement evidence from trials that require ablative dose prescription.

From a clinical perspective, the pattern observed in this cohort has practical implications for patient selection and treatment intent. Because these patients were unfit for standard ablative SBRT owing to poor performance status or proximity of lesions to critical structures, they represent a population in which aggressive dose escalation carries a higher risk of toxicity. In our cohort, local control was favorable (1- and 2-year local recurrence-free survival of 89.7% and 86.9%), whereas event-free survival was short (median 7.2 months) and overall survival was limited (median 30.0 months). Thus, while SBRT effectively addressed the targeted lesions, it did not alter the systemic trajectory that ultimately determined outcomes. This dissociation suggests that, in this setting, durable local control can be achieved without ablative dosing, whereas the overall disease course is dominated by systemic progression. Moreover, moderate-dose SBRT was well tolerated, with all toxicities limited to grade 1–2 and no grade ≥ 3 events. These findings support a pragmatic strategy in which moderate-dose SBRT provides sufficient local control while minimizing the risk of organ-at-risk toxicity and avoiding interruption of systemic therapy, rather than pursuing ablative dose thresholds that may not alter the predominantly systemic course of disease.

With the expanding role of metastasis-directed therapy in OMD, SBRT has become increasingly used. Prior studies have demonstrated improvements in PFS and OS with local therapy. In the phase II SABR–COMET trial, patients with one to five metastases were randomized to standard palliative RT or SABR to all lesions plus systemic therapy. SABR significantly improved OS, with a median OS of 41 months versus 28 months in the initial analysis, and a 22-month benefit in long-term follow-up (28 vs. 50 months; HR 0.47). However, grade ≥ 2 toxicities were more frequent in the SABR arm (29% vs. 9%), and the study included heterogeneous primary tumor types rather than focusing on lung cancer [6,14]. Consistent results have been reported in oligometastatic lung cancer; the randomized trial by Gomez et al. demonstrated improved outcomes with local consolidative therapy, including longer median PFS (14.2 vs. 4.4 months) and OS (41.2 vs. 17.0 months) [15].

Surgical resection, including pulmonary metastasectomy, is also recognized as an established component of metastasis-directed therapy for oligometastatic NSCLC [7]. Although prospective head-to-head comparisons between SBRT and surgical resection are lacking, in colorectal cancer, for which the lung is a common site of metastasis, the role of pulmonary metastasectomy continues to be debated, and metastasis-directed radiotherapy such as SBRT is increasingly considered a less invasive alternative for selected patients [16]. How best to select between metastasis-directed radiotherapy and surgical resection for an individual patient remains an open question, and emerging artificial intelligence and deep learning approaches may eventually help to inform such treatment decisions. This remains an area requiring further investigation. SBRT is generally considered a feasible option for patients who are fragile, elderly, or otherwise unsuitable for surgery. As a non-invasive modality applicable to lesions across varied anatomical sites, SBRT can also be delivered with minimal interruption to ongoing systemic therapy [17]. Our cohort could correspond, in part, to this clinical population, in which moderate-dose SBRT was selected as a treatment option for patients considered unsuitable even for ablative dosing.

The confirmation of active oligometastatic disease before SBRT is a recognized challenge in clinical practice. Imaging-based assessment of oligometastatic disease has inherent limitations, including the recognized false-negative rate of FDG PET-CT for nodal staging. However, per-lesion re-biopsy in oligometastatic disease, particularly of mediastinal or hilar nodes requiring invasive procedures such as endobronchial ultrasound or mediastinoscopy, is impractical and carries non-trivial procedural risk. Imaging-based confirmation by CT and FDG PET-CT, interpreted with RECIST 1.1 criteria [13] and supplemented by tissue biopsy in cases of diagnostic uncertainty, has therefore been adopted as the standard eligibility framework in major oligometastatic NSCLC trials [6,12,15], and in the 2023 ASTRO/ESTRO clinical practice guideline [7].

Presently, there is no consensus regarding the optimal SBRT dose for pulmonary oligometastases. SBRT dose–fractionation schedules vary widely, although prior evidence supports delivery of a BED_10_ ≥ 100 Gy to achieve high LC across multiple metastatic sites, including the lung, liver, and adrenal gland [18,19,20,21]. Despite randomized data supporting SBRT as an effective and safe metastasis-directed therapy [22], delivering ablative doses is often constrained by organ-at-risk limits, proximity to critical structures such as the ultracentral thorax, or prior irradiation [8,9,10,23]. Additional limitations arise at abdominal sites, where SBRT-related toxicities, including radiation-induced liver disease, hepatobiliary injury, gastrointestinal ulceration or perforation, adrenal insufficiency, and dose-dependent renal dysfunction, have been reported [24,25]. These thoracic and abdominal toxicities may necessitate treatment interruptions or schedule modifications, potentially delaying systemic therapy and complicating clinical management.

In contrast, conventional palliative RT is primarily intended for symptom relief rather than durable in-field tumor control and is commonly delivered using schedules such as 20 Gy in 5 fractions or 30 Gy in 10 fractions. Systematic reviews and guideline statements indicate that thoracic palliative RT improves symptoms, whereas longer or higher-dose palliative regimens have not consistently produced superior palliation or survival, outcomes that remain largely determined by systemic disease burden [26,27].

Accordingly, prospective studies in NSCLC have demonstrated the clinical activity of moderate-dose, metastasis-directed RT. In a single-arm phase II trial by Iyengar et al., patients with stage IV NSCLC and ≤6 extracranial lesions who progressed after platinum-based chemotherapy received SBRT to all sites using “equipotent” schedules (19–24 Gy in 1 fraction, 27–33 Gy in 3 fractions, or 35–40 Gy in 5 fractions) delivered concurrently with and followed by erlotinib until progression. Despite mutation-unselected enrollment (no EGFR mutations among those tested), median PFS and OS were 14.7 and 20.4 months, respectively [11]. In a subsequent randomized phase II trial by the same group, patients with limited metastatic NSCLC without EGFR/ALK targetable alterations who achieved PR/SD after 4–6 cycles of platinum-based induction chemotherapy were randomized to maintenance chemotherapy alone or consolidative SBRT to all gross disease followed by maintenance chemotherapy. The trial was stopped early after interim analysis demonstrated significantly improved PFS with consolidative SBRT (median 9.7 vs. 3.5 months; HR 0.304) and comparable toxicity [12]. Our planning and delivery workflow can be benchmarked against these prospective protocols and current guidelines. The dose–fractionation schedules used in our cohort, most commonly 35 Gy in 5 fractions, fall within the equipotent range of 35 to 40 Gy in 5 fractions employed in these trials [11,12]. In addition, simulation with 4D-CT, treatment delivery with VMAT or IMRT, and daily cone-beam CT image guidance are consistent with the practice recommended for metastasis-directed radiotherapy in the 2023 ASTRO/ESTRO clinical practice guideline [7].

Beyond feasibility, moderate hypofractionation may offer advantages in the immunotherapy era. A secondary analysis of KEYNOTE-001 showed that patients with advanced NSCLC who received prior RT experienced longer PFS and OS with pembrolizumab than those without prior RT, suggesting a clinically relevant interaction between RT and PD-1 blockade [28]. From a dose-fractionation perspective, preclinical data indicate that very high single-fraction doses (approximately >12–18 Gy per fraction) can induce TREX1 and reduce RT-induced immunogenicity, supporting the rationale for moderately hypofractionated regimens [29]. Consistent with this concept, the PEMBRO-RT phase II randomized trial used SBRT of 8 Gy × 3 fractions to a single lesion before pembrolizumab, providing clinical precedent for an approximately 7–8 Gy per fraction approach in this setting [30]. Mechanistically, radiation-induced accumulation of cytosolic double-stranded DNA activates the cGAS-STING pathway and drives type I interferon production, whereas induction of the exonuclease TREX1 at higher doses per fraction degrades this substrate and attenuates the immunogenic response [29]. The fractional doses used in our cohort, most commonly 7 Gy, lie below the reported threshold for TREX1 induction and are close to the 8 Gy schedule tested in PEMBRO-RT [30]. Whether moderate-dose SBRT acts synergistically with immune checkpoint blockade in oligometastatic lung cancer cannot be determined from our data, because concurrent systemic therapy was heterogeneous and radiation was delivered with local intent. These considerations nonetheless provide a mechanistic rationale for prospective evaluation of moderate-dose regimens combined with immunotherapy.

Moderate-dose, risk-adapted SBRT is also particularly relevant for nodal metastases, which frequently lie close to central organs at risk and therefore often cannot receive standard ablative doses. Although data specific to nodal oligometastases remain relatively limited compared with pulmonary or osseous sites, several series have reported favorable local control using moderate-dose schedules. In a large mono-institutional series, Caivano et al. reported durable LC after SBRT for nodal metastases in 174 patients with 229 nodal lesions, with lung cancer as the most common primary tumor (31%). Treatment was delivered predominantly with moderate hypofractionation (most commonly 30 Gy in 5 fractions), achieving 1-, 3-, and 5-year LC rates of 93%, 86%, and 86%, respectively [31]. Similarly, Shahi et al. applied risk-adapted planning for SBRT to mediastinal and hilar nodal metastases, which are anatomically challenging because of proximity to central organs at risk. SBRT was most commonly delivered in five fractions (median dose, 35 Gy), resulting in a 2-year cumulative incidence of local failure of 9% [32]. Interest in moderate-dose, five-fraction SABR for patients unfit for standard treatment has also extended to the locally advanced setting. In a recent prospective trial, Arcidiacono et al. treated 50 patients with unresectable locally advanced NSCLC unfit for concurrent chemoradiation using five-fraction SABR (45 Gy to the tumor and 40 Gy to nodal regions), reporting favorable local control with no grade 3 or higher late toxicity [33]. That study also identified a SABR dose below 40 Gy as a predictor of local recurrence. Although that prospective trial differs from our retrospective analysis in both design and disease setting, addressing locally advanced rather than oligometastatic disease, its findings reflect the growing interest in this dose range; its dose–response observation cannot be directly applied to our setting but suggests that dose escalation may warrant consideration in future studies.

In our cohort, lymph node metastases accounted for 38 of 55 treated lesions (69.1%), and local control differed markedly by site (Table 3, Figure 2a). The 2-year local control rate was 95.5% for lymph node metastases, compared with 56.2% for lesions of the lung and chest wall (log-rank *p* = 0.002), and non-lymph node location was associated with local recurrence on univariable analysis (HR 6.008; 95% CI, 1.165–30.976; *p* = 0.032), although this association did not persist after multivariable adjustment (Table 4). Our nodal outcomes fall within the range reported in the series described above, in which 1- and 3-year local control rates of 93% and 86% were achieved with predominantly moderate hypofractionation [31], and the 2-year cumulative incidence of local failure was 9% after risk-adapted planning for mediastinal and hilar nodes [32]. All five adrenal lesions also remained controlled, although this number is too small for meaningful interpretation. Previous adrenal series have generally employed ablative doses to achieve high local control [19], and whether moderate-dose schedules are sufficient at this site requires further study. These findings suggest that moderate-dose schedules may provide durable control of nodal disease, whereas pulmonary parenchymal lesions may require higher doses. Rather than adherence to a uniform ablative threshold, dose tailoring based on lesion location, organ-at-risk proximity, and patient-related factors may represent a practical basis for individualizing prescription in routine clinical practice.

Local control did not differ significantly by delivered dose within this cohort. The 2-year local control rate was 90.9% for lesions treated with a BED10 of 59.5 Gy or more and 74.6% for those treated below this threshold (log-rank *p* = 0.245, Figure 2b), and low dose was not associated with local recurrence on univariable analysis (HR 2.403, 95% CI 0.525–11.012, *p* = 0.259). The direction of this difference is consistent with the dose–response relationship reported in trials employing higher doses [33], but the narrow dose range in this study, in which most lesions received 35 Gy in 5 fractions, together with the small number of recurrence events, limits any inference about an optimal threshold. Local control within this dose range therefore appeared relatively insensitive to small differences in prescribed dose.

Outcomes also varied according to oligometastatic subtype. Local control was maintained in all 13 lesions treated in patients with de novo disease, whereas the 2-year rate was 82.8% in those with induced oligometastatic disease (log-rank *p* = 0.247, Figure 2c). The difference was more evident for overall survival, with death occurring in 2 of 10 patients with de novo, 1 of 8 with repeat, and 17 of 25 with induced disease. The 2-year overall survival rate was 62.5% for de novo compared with 48.2% for induced disease (log-rank *p* = 0.050). A history of polymetastatic disease also showed the largest effect estimate in the multivariable model for local recurrence (HR 8.219, 95% CI 0.890–75.880, *p* = 0.063, Table 4), although the confidence interval was wide. These observations are in keeping with the prognostic intent of the ESTRO/EORTC classification [1], in which induced oligometastatic disease reflects a previously higher metastatic burden, and they suggest that oligometastatic subtype may be worth considering when weighing the expected benefit of metastasis-directed treatment.

The use of 18F-FDG PET/CT in radiation therapy planning for lung cancer has been recently addressed in joint practice recommendations by the European Association of Nuclear Medicine, the Society of Nuclear Medicine and Molecular Imaging, and the European Society for Radiotherapy and Oncology, which highlight its value in improving delineation of metabolically active tumor volumes, differentiating tumor from atelectasis, and identifying additional disease that may modify the target volume [34]. In the oligometastatic setting in particular, accurate metabolic characterization is valuable for confirming the number and extent of active lesions and for distinguishing metabolically active disease from post-treatment or inflammatory changes, thereby supporting appropriate target selection. In the present study, PET/CT was used for staging and lesion identification but was not routinely co-registered with the simulation CT for target volume delineation. Incorporating routine PET/CT co-registration into the planning workflow, in line with these joint recommendations, represents a concrete direction for refining our protocol in future practice.

The most common toxicity in our cohort was radiation pneumonitis (9.3%), all of grade 1–2, with no grade ≥ 3 events observed. Distinguishing radiation pneumonitis from systemic therapy-related pneumonitis, including immune checkpoint inhibitor (ICI)-related pneumonitis, was based on the temporal relationship to SBRT, anatomic correspondence to the radiation field, and CT findings. Radiation pneumonitis typically presents as focal consolidation or ground-glass opacities within or adjacent to the high-dose region, in contrast to the bilateral, multilobar pattern more commonly seen in ICI-related pneumonitis [35,36].

This study has several limitations. First, as a retrospective, single-institution analysis, it is subject to selection bias, variability in clinical decision-making, and the inherent constraints of retrospective data collection. In addition, the median follow-up of 14.7 months is relatively short, and late local recurrence and delayed radiation toxicities may not have been fully captured. The small sample size may also have limited the precision of time-to-event estimates. Furthermore, with only 55 lesions and seven local recurrence events in the analytic dataset, the precision of subgroup comparisons and multivariable estimates is constrained. The wide confidence intervals around the hazard ratios in Table 4 reflect this limitation, and observed associations should be regarded as exploratory and hypothesis-generating rather than confirmatory. Nonetheless, lung cancer–specific reports evaluating moderate-dose, metastasis-directed SBRT remain limited, particularly in cohorts enriched for nodal disease and treated under real-world organ-at-risk constraints, and our findings may therefore inform risk-adapted practice when ablative dosing is not feasible. Second, patients were treated in complex clinical contexts with heterogeneous systemic therapies and individualized dose–fractionation schedules dictated by target location and organ-at-risk limitations, making it difficult to isolate the independent contribution of SBRT to survival and failure patterns. However, as a preliminary, exploratory experience, such heterogeneity reflects how moderate-dose SBRT is applied in routine practice, and describing outcomes across these regimens may help inform dose selection in future studies. Third, although no grade ≥ 3 toxicities were observed, retrospective toxicity assessment may underestimate low-grade or late adverse events, highlighting the need for larger cohorts, longer follow-up, and prospective validation. Fourth, quantitative PET metabolic parameters, such as maximum standardized uptake value and metabolic tumor volume, were not collected in our database. PET-CT was not performed in all patients, and imaging protocols were not uniform across the multi-year study period. For these reasons, the predictive value of metabolic parameters for local recurrence could not be assessed in this cohort, and this question warrants evaluation in future studies with standardized imaging protocols. Finally, patients with grossly evident invasion of the great vessels, including the superior vena cava, were not represented in this cohort. As our moderate-dose regimens were largely selected to address proximity to critical structures or organ-at-risk constraints rather than overt vascular invasion, our findings should not be extrapolated to such scenarios, in which dose fractionation, treatment intent, and safety considerations may substantially differ.

## 5. Conclusions

In this preliminary single-center experience, moderate-dose SBRT achieved favorable local control with minimal toxicity in patients unfit for standard ablative SBRT. Distant progression was the predominant pattern of failure, whereas local failure was uncommon. When ablative dosing cannot be safely delivered, a risk-adapted moderate-dose strategy offers a clinically reasonable option that can provide durable local control while preserving safety.

## Figures and Tables

**Table 1:** Patient characteristics.

Variable	*N* (Total = 43)	(%)
Age, mean (range)	66	(43–85)
Gender		
Male	31	(72.1)
Female	12	(27.9)
Histology		
Adenocarcinoma	19	(44.2)
Squamous cell	9	(20.9)
Mixed small and squamous cell	1	(2.3)
Non-small cell	3	(7.0)
Pleomorphic	1	(2.3)
Poorly differentiated	1	(2.3)
Small cell	9	(20.9)
Timing of SBRT from initial diagnosis		
Within 6 months	9	(20.9)
More than 6 months	34	(79.1)
Current Systemic Treatment		
Yes	30	(69.8)
No	13	(30.2)
Oligometastatic state at SBRT *		
Induced OMD	25	(58.1)
Repeat OMD	12	(27.9)
De novo OMD	6	(14.0)
Currently progressing lesion at SBRT		
Yes	37	(86.0)
No	6	(14.0)
Number of lesions		
1	34	(79.1)
2	6	(14.0)
3	3	(7.0)

Abbreviations: NSCLC, non-small cell lung cancer; NOS, not otherwise specified; SCLC, small cell lung cancer; ECOG, Eastern Cooperative Oncology Group; OMD, oligometastatic disease; SBRT, stereotactic body radiation therapy. * Oligometastatic states are classified according to the ESTRO/EORTC consensus [1].

**Table 2:** Lesion-level characteristics (*N* = 55 lesions in 43 patients).

Variable	*N* (Total = 55)	(%)
Site of metastasis		
Lymph Node	(total = 38)	(total = 69.1)
Hilar/Peribronchial	3	(5.5)
Mediastinal/Subcarinal	19	(34.5)
Supraclavicular	5	(9.1)
Neck node	1	(1.8)
Axillary LN	3	(5.5)
Abdominal LN	7	(12.7)
Non-lymph node	(total = 17)	(total = 30.9)
Adrenal	5	(9.1)
Lung	10	(18.2)
Chest wall	2	(3.6)
Dose/Fraction		
35 Gy in 5 fractions	35	(63.6)
30 Gy in 5 fractions	10	(18.2)
35 Gy in 7 fractions	4	(7.3)
40 Gy in 5 fractions	3	(5.5)
25 Gy in 5 fractions	1	(1.8)
36 Gy in 4 fractions	1	(1.8)
36 Gy in 6 fractions	1	(1.8)

Abbreviations: LN, lymph node.

**Table 3:** Stratified analyses of local control.

Stratification	Lesions	Recurrences	1-Year (%)	2-Year (%)	*p*-Value
Metastatic site					0.002
Lymph node	38	2	100	95.5 (87.1–100)	
Lung and chest wall	12	5	56.2 (33.6–94.3)	56.2 (33.6–94.3)	
Adrenal	5	0	100	100	
BED_10_					0.245
≥59.5 Gy	39	4	94.6 (87.5–100)	90.9 (81.5–100)	
<59.5 Gy	16	3	74.6 (52.1–100)	74.6 (52.1–100)	
Oligometastatic subtype					0.247
De novo	13	0	100	100	
Repeat	9	1	100	NE	
Induced	33	6	82.8 (70.0–98.0)	82.8 (70.0–98.0)	

All toxicities were graded according to the Common Terminology Criteria for Adverse Events; no grade ≥ 3 events were observed.

**Table 4:** Factors affecting local recurrence after radiotherapy.

	Univariate Analysis			Multivariate Analysis		
Variable	HR	95% CI	*p*-Value	HR	95% CI	*p*-Value
Age						
<65	Ref			Ref		
≥65	5.343	0.992–28.772	0.051	4.151	0.516–33.380	0.181
Gender						
Male	Ref					
Female	1.897	0.423–8.507	0.402			
Histology						
Adenocarcinoma	Ref					
Non-adenocarcinoma	0.658	0.147–2.945	0.584			
History of polymetastasis						
No	Ref			Ref		
Yes	4.327	0.517–36.242	0.177	8.219	0.890–75.880	0.063
>6 months from initial diagnosis						
No	Ref					
Yes	1.351	0.162–11.246	0.781			
Number of lesions						
1	Ref					
≥2	0.303	0.036–2.519	0.269			
Site of metastasis						
Lymph node	Ref			Ref		
Non-lymph node	6.008	1.165–30.976	0.032	4.257	0.645–28.100	0.133
Dose						
BED_10_ ≥ 59.5 Gy	Ref					
BED_10_ < 59.5 Gy	2.403	0.525–11.012	0.259			

Abbreviations: HR, hazard ratio; CI, confidence interval; BED_10_, biologically effective dose calculated with an α/β ratio of 10 Gy. A BED_10_ of 59.5 Gy corresponds to 35 Gy in 5 fractions.

**Table 5:** Treatment-related toxicities.

Toxicity	Grade 1	Grade 2	Grade ≥ 3	Total, *n* (%)
Radiation pneumonitis	1	3	0	4 (9.3)
Dermatitis	1	1	0	2 (4.7)
Mucositis	1	0	0	1 (2.3)
Esophagitis	0	1	0	1 (2.3)
Any toxicity	3	5	0	8 (18.6)

All toxicities were graded according to the Common Terminology Criteria for Adverse Events; no grade ≥ 3 events were observed.

## Data Availability

Data are not publicly available due to ethical restrictions. Further inquiries can be directed to the corresponding author.

## Abbreviations

The following abbreviations are used in this manuscript:

BED_10_	Biologically effective dose at α/β = 10 Gy
CI	Confidence interval
CT	Computed tomography
4D-CT	Four-dimensional computed tomography
DDFS	Disseminated disease-free survival
ECOG	Eastern Cooperative Oncology Group
EFS	Event-free survival
GTV	Gross tumor volume
HR	Hazard ratio
IMRT	Intensity-modulated radiation therapy
ITV	Internal target volume
LC	Local control
LN	Lymph node
LRFS	Local recurrence-free survival
NSCLC	Non-small cell lung cancer
OMD	Oligometastatic disease
OS	Overall survival
PET/CT	Positron emission tomography/computed tomography
PFS	Progression-free survival
PR	Partial response
PTV	Planning target volume
RECIST	Response Evaluation Criteria in Solid Tumors
RT	Radiation therapy
SBRT	Stereotactic body radiation therapy
SCLC	Small cell lung cancer
SCL	Supraclavicular
VMAT	Volumetric-modulated arc therapy
